# The complete mitochondrial genome of *Platycephalus* sp.1 (Teleostei, Platycephalidae) obtained by whole genome sequencing

**DOI:** 10.1080/23802359.2021.1937361

**Published:** 2021-06-07

**Authors:** Hao Zhang, Keyue Shen, Shiyan Feng, Chenchen Wang, Shengyong Xu

**Affiliations:** Fisheries College, Zhejiang Ocean University, Zhoushan, China

**Keywords:** Platycephalidae, mitogenome, *Platycephalus* sp.1, whole genome sequencing

## Abstract

In this study, we used next-generation sequencing to obtain the complete mitochondrial genome of *Platycephalus* sp.1. This mitochondrial genome, consisting of 16,552 base pairs (bp), contains 13 protein-coding genes, two ribosomal RNAs, 22 transfer RNAs, and two non-coding control regions (control region and origin of light-strand replication) as those found in other vertebrates. Control region, of 877 bp in length, is located between tRNA^Pro^ and tRNA^Phe^. Within the control region, typical conserved domains, such as the termination-associated sequence, central and conserved sequence blocks domains were identified. The overall base composition shows 25.83% of T, 29.98% of C, 27.01% of A, and 17.18% of G, with a slight A + T rich feature (52.84%). The complete mitogenome data provides useful genetic markers for the studies on the molecular identification, population genetics, phylogenetic analysis and conservation genetics.

The flathead fish, *Platycephalus* sp.1 has been long recognized in the coastal waters of Japan with the Japanese name Yoshino-gochi (Kamei and Ishiyama 1969; Nakabo 2002). Recently, this species was shown to be morphologically and genetically distinct and also found in the Chinese coastal waters (Qin et al. 2013; Chen et al. 2020). Since its relatively recent discovery, more genetic and genomic information of *Platycephalus* sp.1 are needed. The mitochondrial genome sequence provides valuable molecular markers for phylogenetic studies. At present, only limited mitochondrial sequences have been published for *Platycephalus* sp.1. To increase our understanding and provide useful genetic resources of *Platycephalus* sp.1, we present the complete mitochondrial genome of this species and analyze the mitochondrial genome with other congeners of suborder Platycephaloidei in this study.

The sample of *Platycephalus* sp.1 was collected from the coastal water of Zhoushan (30.11°N, 122.37°E), China in September 2019. The examined specimen was preserved at Fisheries Ecology and Biodiversity Laboratory in Zhejiang Ocean University under specimen accession NO. ZJOU-04079. The genomic DNA was extracted from dorsal-lateral muscles (30 mg) using Rapid Animal Genomic DNA Isolation Kit (Sangon Biotech Co., Ltd., Shanghai, CN). Whole genome sequencing (sequencing depth 10X) was conducted using an Illumina Hiseq 4000 with the sequencing insertion of 350 bp. Quality check for sequence data was done using FastQC (Andrews 2010) and the data were filtered using Trimmomatic 0.36 (Bolger et al. 2014) with default parameters. The filtered clean data were assembled and mapped to the complete mitogenome sequence using NOVOPlasty v3.7.2 (Dierckxsens et al., 2017) with default settings. Subsequently, the assembled sequence was annotated using the online Mitochondrial Genome Database of Fish server (http://mitofish.aori.u-tokyo.ac.jp/, Iwasaki et al. 2013) and the MITOS Web Server (http://mitos.bioinf.uni-leipzig.de/index.py, Bernt et al. 2013).

The final sequence has been deposited in GenBank with accession number MT584655. The complete mitochondrial genome of *Platycephalus* sp.1 (16,552 bp in length) consists of 13 protein-coding genes, 22 tRNA genes, two rRNA genes, and two non-coding control regions (control region and origin of light-strand replication). The arrangement of all genes is identical to those of most vertebrates (Chen, 2013; Chiang et al., 2013; Wang et al., 2008). Most of the genes are encoded on the heavy strand (H-strand), except for the eight tRNA genes (-Gln, -Ala, -Asn, -Cys,-Tyr, -Ser, -Glu and -Pro) and one protein-coding gene (ND6). The overall base composition is 25.83% of T, 29.98% of C, 27.01% of A and 17.18% of G, with a slight A + T-rich feature (52.84%). Except for COI and ND4 starting with GTG, the remaining 11 protein-coding genes start with ATG. It is important to note that some of the protein-coding genes (7 of 13 genes) are inferred to terminate with an incomplete stop codon (COII, COIII, ND2, ND3, ND4, ATPase 6 and Cyt *b*), with five (COI, ATPase8, ND4L, ND5 and ND6) sharing TAA and one (ND1) using TAG as a stop codon, respectively. These features are common among vertebrate mitochondrial genomes, and TAA is thought to have evolved via posttranscriptional polyadenylation (Ojala et al. 1981). The non-coding control region (D-loop) is 877 bp in length, and is located between tRNA^Pro^ and tRNA^Phe^. Within the D-loop, a termination-associated sequence (TAS), conserved sequence blocks (CSB-1, CSB-2 and CSB-3), and several areas of highly conserved sequence (CSB-F, CSB-E and CSB-D) were detected. The two ribosomal RNA genes, 12S rRNA (951 bp) and 16S rRNA (1695 bp), are located between tRNA^Phe^ and tRNA^Leu^.

Phylogenetic relationships were constructed using Maximum Likelihood (ML) implemented in MEGA 6 (Tamura et al. 2013) for 6 species within the suborder Platycephaloidei based on 13 mitochondrial protein-coding genes (Figure 1(a)). This phylogenetic tree showed that *Platycephalus* sp.1 has a relatively close relationship with *Platycephalus indicus*. Considering the lack of complete mitochondrial genome sequences of flatheads, and to further determine the taxonomic status of *Platycephalus* sp.1, the phylogeny of 15 species in the genus *Platycephalus* were reconstructed using ML based on the mitochondrial gene COI (Figure 1(b)). The results also suggested that there is a large evolutionary divergence between *Platycephalus* sp.1 and other flathead congeners, which could strongly support the validity of *Platycephalus* sp.1 at genetic level. More complete mitochondrial genome sequences are needed in future studies for more robust phylogenetic analyses of flatheads. The information of the mitogenome will be useful for future phylogenetic studies and specimen identification of Platycephalidae species.

**Figure 1.**
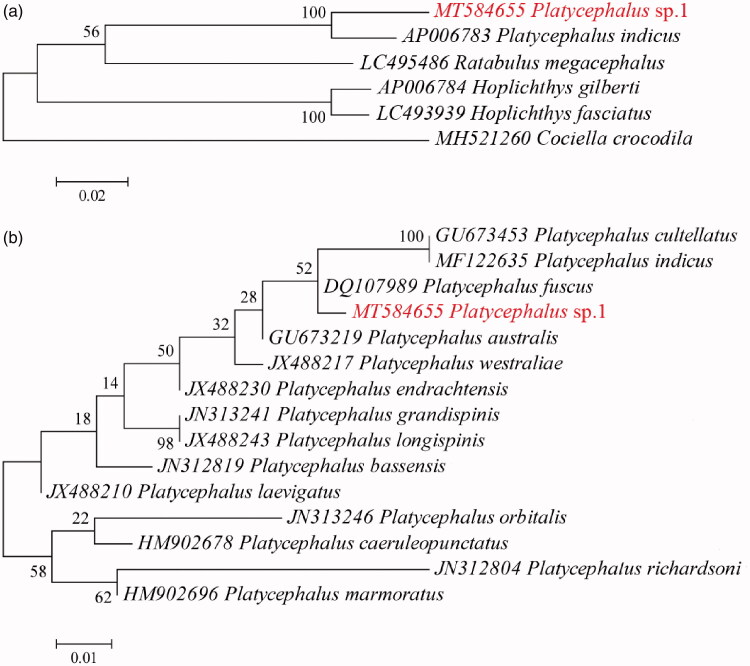
Phylogenetic topologies constructed in this study. (a) Maximum likelihood (ML) tree for 6 species of suborder Platycephaloidei based on 13 mitochondrial protein-coding genes; (b) ML tree for 15 species of genus *Platycephalus* based on mitochondrial COI gene fragments.

## Data Availability

The data that support the findings of this study is openly available in GenBank of NCBI at https://www.ncbi.nlm.nih.gov under accession number MT584655.
